# Optimizing Medication Access and Use in Inflammatory Bowel Disease: The Roles and Impact of Clinical Pharmacists and Pharmacy Technicians

**DOI:** 10.1093/crocol/otad014

**Published:** 2023-03-09

**Authors:** Shubha Bhat

**Affiliations:** Department of Pharmacy, Cleveland Clinic Foundation, Cleveland, Ohio, USA; Digestive Disease & Surgery Institute, Cleveland Clinic Foundation, Cleveland, Ohio, USA

Inflammatory bowel disease (IBD), a relapsing chronic inflammatory disease of the gastrointestinal tract, is anticipated to affect 3 million adults in the United States.^1^ However, the prevalence of IBD may be greater, as Ye et al identified an increase in IBD diagnoses by 123% over 9 years.^2^ Presently, there are several treatment options available for IBD management, including biologics and small molecules.^3,4^ Despite representing a milestone in IBD management, these treatments are considered a significant driver of IBD-related direct costs due to their price.^5^ Given the growing IBD prevalence and use of effective, but costly pharmacotherapies, many payors have implemented prior authorization and step therapy processes in place to help control healthcare-related expenditure. Unfortunately, these processes have led to increased burdens and medication-related access challenges.^6^ For example, prior authorizations may not be reviewed by payors in a timely manner or may be denied and subjected to several levels of appeals, ultimately leading to delayed treatment initiation and/or changes to the original treatment plan. Alternatively, insurance coverage for the requested medication may be approved, but may not be financially feasible for the patient, necessitating the identification of other venues to assist with medication affordability and access.

Choi et al highlight the prevalence of processes relating to IBD medication access, noting that of the 1800 referrals received by the tertiary academic institution’s specialty pharmacy to initiate, dose adjust, or continue biologic or small molecule treatment, 94% required a prior authorization.^7^ Unfortunately, 297 prior authorization requests were denied, with 77% being approved after 1 round of appeals, and the remaining requiring a second or third level of appeal. Of all the referrals, only 2% had to switch to an alternative treatment. Additionally, 69 patients were enrolled into a manufacturer patient assistance program. Unique to this manuscript, however, is the description of the clinical pharmacy team’s role and impact in the prior authorization service and patient access to treatment. Consisting of (1) IBD clinical pharmacists, who primarily provided appeal and coordination of care services once authorization approval was obtained, such as pending prescriptions for signature with correct pharmacy selected for e-prescribing and medication education, and (2) pharmacy technicians, who completed benefits investigation, submitted prior authorization, and identified patient assistance programs, the clinical pharmacy team was able to design and execute a streamlined and efficient workflow that resulted in 98% of patients successfully starting on the intended IBD therapy.

Given that the IBD medication arsenal is projected to grow with more complex therapies and medication access barriers are anticipated to remain prevalent, IBD centers should consider embedding or partnering with a clinical pharmacy team.^8^ These teams may be housed within a specialty pharmacy, work directly in the IBD clinic, or participate in a hybrid model where time is split between the specialty pharmacy and IBD clinic. Clinical pharmacists complete graduate education to earn a Doctor of Pharmacy (PharmD) degree and post-graduate training to further their pharmacology skillset and clinical experiences. Pharmacy technicians, who can obtain certifications, work closely with the clinical pharmacists to assist with medication access and distribution. Given these collective backgrounds and experiences, clinical pharmacy teams are well positioned to assist with medication-related needs, including education, monitoring, and access.

Choi et al also outline additional clinical interventions provided by the IBD pharmacists beyond preventing treatment lapses and providing patient education, noting that inclusion of IBD pharmacists in patient care allows for additional touchpoints to help facilitate refills and appropriate follow-ups for medication monitoring and evaluation. Embedding clinical pharmacists with a collaborative scope of practice into the IBD clinic would permit for greater outreach and involvement in patient care, as pharmacists are well equipped and positioned to: provide medication education including device teaching, facilitate shared decision making, participate and/or drive medication monitoring by ordering and interpreting labs and making necessary adjustments to treatment under a gastroenterologist’s supervision, manage adverse effects, provide smoking cessation services, ensure patients are up to date on health maintenance recommendations, and assist with formulary management.^9–11^ Future studies should focus on these additional roles of IBD clinical pharmacists and effect on patient care and outcomes.

The impact of IBD on the healthcare system is only projected to increase given the growing prevalence of this condition and expanding arsenal of effective but costly pharmacotherapies. As a result, the utilization of an IBD multidisciplinary team, consisting of gastroenterologists, colorectal surgeons, advanced practice providers, nurses, dietitians, and psychologists/psychiatrists, to ensure holistic management and improve patient outcomes is becoming a standard model of care.^12^ Inclusion of clinical pharmacy teams in this model should be a best practice consideration, given the involvement of complex and costly IBD medications. Moreover, these medications require precise monitoring, as medication nonadherence, inadequate and delayed treatment access, and suboptimal dosing all negatively affect disease control and patients’ quality of life. However, one potential challenge that IBD centers may face with embedding or partnering with clinical pharmacy teams is the lack of data highlighting the return on investment with these positions. Despite pharmacists and pharmacy technicians showcasing their ability to optimize medications in several ways, including access and use, a financial analysis is often a key component of position justification. Future research is needed to evaluate both the clinical and economic value of IBD clinical pharmacy teams.

## Data Availability

NA—editorial article.

## Summary

Clinical pharmacy teams can optimize medication access and use and should be integrated in inflammatory bowel disease centers.
